# Real-world data on cervical cancer risk stratification by cytology and HPV genotype to inform the management of HPV-positive women in routine cervical screening

**DOI:** 10.1038/s41416-020-0790-1

**Published:** 2020-04-03

**Authors:** Dana Hashim, Birgit Engesæter, Gry Baadstrand Skare, Philip E. Castle, Tone Bjørge, Ameli Tropé, Mari Nygård

**Affiliations:** 1 Section for cervical cancer screening, Cancer Registry of Norway, Oslo, Norway; 2 Department of Epidemiology and Population Health, Albert Einstein College of Medicine, Bronx, NY USA; 3 Department of Global Public Health and Primary Care, University of Bergen, Bergen, Norway; 4 Department of Research, Cancer Registry of Norway, Oslo, Norway

**Keywords:** Cancer screening, Cancer prevention

## Abstract

**Background:**

HPV16/18 detection may improve cervical cancer risk stratification and better guide which HPV-positive women warrant immediate colposcopy/biopsy. We estimated risks of cervical precancer and cancer by HPV genotype and cytology during the implementation phase of primary HPV testing in Norway.

**Methods:**

A total of 3111 women, aged 34–69 years, testing HPV-positive at baseline and undergoing cytology testing from February 2015 to April 2018 had data available for analysis. Risk estimates with 95% confidence intervals (95%CIs) of cervical intraepithelial neoplasia grade 3 or more severe (CIN3+) were estimated for cytology results and HPV genotypes (HPV16, HPV18, and other high-risk HPV).

**Results:**

CIN3+ risks were higher for HPV16/18 than other high-risk HPV genotypes. Among women with any cytologic abnormality [atypical squamous cells of undetermined significance or worse], immediate risks were 57.8% (95%CI = 53.0–62.6%) for HPV16, 40.2% (95%CI = 32.3–49.2%) for HPV18, and 31.4% (95%CI = 28.7–34.3%) for other high-risk HPV. Among those with normal cytology, CIN3+ risks were 19.9% (95%CI = 15.0–26.1%) for HPV16 positives, 10.8% (95%CI = 5.6–20.5%) for HPV18 positives, and 5.5% (95%CI = 4.2–7.1%) for other high-risk HPV.

**Conclusions:**

The benefits and harms of managing women based on HPV positivity and cytology results can be better balanced by inclusion of HPV genotyping in screening and choosing more conservative management for other high-risk HPV compared to HPV16/18.

## Background

Women with human papillomavirus (HPV) infections that persist for at least one^1^ or two years^2^ are at high risk of cervical precancers (cervical intraepithelial neoplasia grade 2 [CIN2], grade 3 [CIN3], adenocarcinoma in situ [AIS] and cervical cancer). HPV infection in women over 30 years old is more likely to be persistent, with a greater likelihood of the development of high-grade cervical intraepithelial neoplasia (CIN2/3) or cervical cancer.^3^

Randomised controlled trials have demonstrated that primary HPV screening leads to a greater reduction in the overall incidence of cervical cancer compared to conventional cytology-based screening and it is now recommended to use HPV as a screening test for women over 30 years of age.^4,5^ HPV testing is also more sensitive than cervical cytology alone in detecting CIN2 or more severe diagnoses (CIN2+) and CIN3 or more severe diagnoses (CIN3+).^5–8^ However, primary HPV screening also detects transient HPV infections, which increases the number of positive screening results and potentially the number of colposcopies performed,^5,9^ the latter depending on the criteria for referral to colposcopy.

Particularly for HPV-positive women with cytology negative for intraepithelial lesion or malignancy (NILM) or low-grade cytology, over-referral to colposcopy can lead to a larger number of colposcopy/biopsy results negative for CIN2+.^10^ This presents a major challenge to the limited colposcopy capacity in most healthcare systems.^10,11^ It is important to choose a colposcopy referral threshold based on the balance between benefits and harms.

The optimal triage protocol for HPV-positive women in routine clinical setting is not yet determined but cytology is currently the general standard. The risk of CIN2+ and CIN3+ varies among oncogenic HPV genotypes.^2,12–15^ HPV16 and/or 18 infection are found in 70% of cervical cancers,^16^ and confer a higher risk than other high-risk HPV genotypes.^17,18^ Among women undergoing primary HPV screening, partial HPV genotyping in combination with cytology may better determine women who should be referred to biopsy compared to cytology alone. In a large US cohort study, women positive for HPV16 were at highest risk for CIN3+, followed by HPV18.^19^ Similar results were also found in a Japanese longitudinal study^20^ and a large clinical trial, ATHENA (Addressing the Need for Advanced HPV Diagnosis).^21^ However, observational studies and randomised controlled trials have specific enrolment criteria that may limit the applicability of results to whole populations. Such limitations include results reflecting subgroups of populations and possible under- or over-estimation of outcomes due to censoring and aggressive follow-up.^21,22^ Risk assessments using real-world or population-based data is needed to ensure “equal management for equal risk” for referral to colposcopy for populations with different patterns of care, compliance, testing, rescreening intervals, and risk factors.^12,23^ Consequently, national screening programs should evaluate and calibrate their screening algorithms based on accepted cervical cancer risk threshold for referring to colposcopy and based on the population’s real-world screening data and capacity.

The Norwegian Cervical Cancer Screening Program (NCCSP) requires data generated within Norway using a real-world population base to modify national screening guidelines. This study aimed to estimate CIN3+ and CIN2+ risks by combinations of HPV16, HPV18, and other high-risk genotypes and cytological results among women in the Norwegian cervical cancer screening population.

## Methods

### Participants

Women aged 34–69 years and living in four Norwegian counties (Sør- and Nord-Trøndelag, Hordaland and Rogaland), were pseudo-randomly assigned by birthday to undergo 5-yearly primary HPV screening or continue to receive 3-yearly cytology screening (even or odd day of birth, respectively), from February 2015 until April 2018.^24^ An exfoliated cervical specimen was collected during pelvic examination using a routinely available collection devices, usually a dry brush for collecting exfoliating cells, which was then rinsed immediately after collection into a ThinPrep (PreservCyt® Solution, Hologic, Inc., Marlborough, MA, USA) for HPV and liquid-based cytology (LBC) testing. Vials were then transported to one of three laboratories for pre-processing and evaluation. All participating laboratories followed a set of guidelines, approved by the Academic Panel for HPV screening implementation in Norway and described in the Quality Assurance Manual for the NCCSP, for allocation, conduction and follow-up of both screening strategies to unify implementation routines.

### Laboratory testing

#### HPV testing

HPV testing was conducted utilising the cobas 4800 HPV test (Roche Diagnostics, Basel, Switzerland) as previously described.^24^ Briefly, the real-time polymerase chain reaction-based method detects 14 HPV genotypes. Results are reported separately for HPV16 and HPV18. The other 12 types (31, 33, 35, 39, 45, 51, 52, 56, 58, 59, 66, and 68) are reported concurrently as a pooled result referred to as other high-risk HPV.^25,26^

#### Cytology testing

For HPV-positive women, a baseline cytological evaluation of the cervical sample was performed at T_0,_ the beginning of the observation period for each subject (Supplementary Fig. 1). Liquid-based cytology was reported according to the Bethesda system^27^ as unsatisfactory, NILM, atypical squamous cells of undetermined significance (ASC-US), low-grade squamous intraepithelial lesion (LSIL), atypical glandular cells of undetermined significance (AGC), atypical squamous cells-cannot exclude HSIL (ASC-H), high-grade squamous intraepithelial lesion (HSIL), adenocarcinoma in situ (AIS) and cervical carcinoma. If the cytology result was ASC-US or more severe interpretation, “immediate” colposcopy and biopsy were recommended.

If the baseline cytology test was NILM, then a 12-month follow-up period was recommended prior to repeating the HPV testing. If the repeat HPV test was positive, women were referred for colposcopy with biopsy. If the repeat HPV test was negative, then women return to routine screening and scheduled to be re-screened by HPV testing in 5 years.^28^

**Fig. 1:**
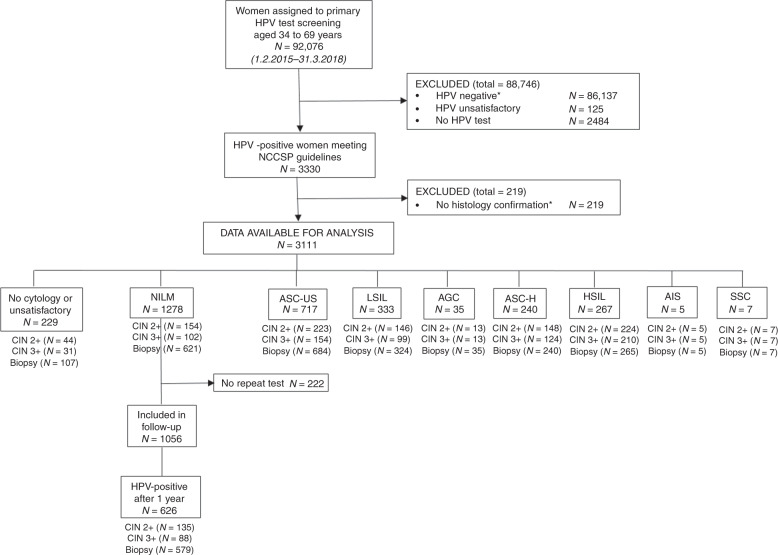
Flow diagram describing selection criteria of primary HPV test participants in the Norwegian Cervical Cancer Screening Program, 2015 to 2018. *HPV negatives (83,601) and those 2536 HPV positives with limited follow-up due to late testing because they had been screened towards the end of the enrolment period. Abbreviations: NILM, negative for intraepithelial lesion or malignancy; ASC-US, atypical squamous cells of undetermined significance; LSIL, low-grade squamous intraepithelial lesions, AGC, atypical glandular cells; ASC-H, atypical squamous cells, cannot rule out high-grade squamous intraepithelial lesion; HSIL, high-grade squamous intraepithelial lesions; ACIS; atypical glandular cells cervical adenocarcinoma in situ; SCC, squamous cell carcinoma.

A unique identification number, allowing identification of every legal resident in Norway, was used to link the information on cytology, HPV tests and histology at an individual level. Relevant screening results were extracted from the databases at the Cancer Registry of Norway for 92,076 women aged 34–69 years who underwent primary HPV screening with no recorded abnormal HPV-tests, cytology or histology (CIN2+) the last two years before the baseline screening test (Fig. 1). A total of 5866 (6.4%) were HPV positive, although 2536 had been excluded due to late testing because they had been screened towards the end of the enrolment period. Exclusions were applied to women with unsatisfactory HPV test results with no repeat HPV test, HPV-negative results, and women with no histological confirmation. HPV negative women were excluded mainly because they require 5 years of follow up and the randomised implementation of HPV primary screening began in 2015. A total of 3111 HPV-positive women had complete data available for analysis that met NCCSP guidelines.

### Use of Norwegian Cancer Registry data for identification and follow-up of study subjects

The Cancer Registry of Norway receives reports of cancer and precancerous lesions with compulsory central registration of cervical cytology, histology, and all HPV test results (both positive and negative), as previously described.^29–31^ Histological diagnoses endpoints were CIN2+ and CIN3+ according to WHO guidelines.^32^

### Statistical analysis

Follow-up started at the date of the baseline cytology test and continued for all women until the worst histologically confirmed lesion was identified. All other observations were censored after the end of recommended follow up (9 months for HPV-positive and ASC-US or worse results or 21 months for HPV-positive and NILM results), which was according to NCCSP guidelines (Supplementary Fig. 1).

Cytology results of AGC, ASC-H, HSIL, AIS and cervical carcinoma were combined into a single “high-grade” category and LSIL and ASC-US were combined into a single “low-grade” category. HPV genotype results were categorised hierarchically according to cancer risk from highest to lowest risk: HPV16 > HPV18 > other high-risk HPV.^33–36^

Given that cervical carcinoma has a low incidence in Norway and the primary goal of cervical screening is to prevent invasive cancer, we used CIN3+ risk as our primary endpoint. We emphasised results for CIN3+ risk rather than CIN2+ because CIN2 is unreliably determined by pathologists^37,38^ and often regresses.^39,40^

CIN3+ and CIN2+ risks with 95% confidence intervals (95%CI) for each screening HPV genotype/cytology result combination were estimated using the Kaplan–Meier failure function.^41^ Risks of CIN2+ and CIN3+ were estimated among women with HPV-positive and ASC-US+ at 9 months and among women with HPV-positive and NILM at 21 months i.e. the baseline CIN3+ (T_0_) for those referred immediately to colposcopy and the subsequent CIN3+ risk for those undergoing follow-up HPV testing for a second time after 12 months, as per NCCSP. In the follow-up after 12 months, 222 (17%) out of 1278 HPV-positive and NILM women did not come to the second round of screening. We also compared risks for younger (35–43 years) and older (44–69 years) women stratified by the median age of the screened women, using the log-rank test of equality for failure functions to test for significant differences by age.^42^ All statistical analyses were done using STATA statistical software: release 15 (College Station, TX: StataCorp LLC).

## Results

### Testing Results

HPV genotype and cytology counts are given for all HPV-positive women, overall and stratified by median age (Table 1). Categorising HPV-positive women hierarchically based on genotype, 650 (20.8%) women were HPV16 positive, 221 (7.1%) were HPV18 positive, and 2148 (69.0%) were positive for other high-risk HPV; and 92 (3.0%) for whom no HPV genotype details were recorded; these were included in the overall HPV positive category. There was an increasing percentage of HPV-positive women with high-grade cytology with increasing cancer risk allocated to the individual HPV genotype (*P*_trend_ < 0.001). Older women were more likely to have a NILM cytology than younger women (*P* < 0.001).

**Table 1:** Primary HPV test by cervical cytology and age group.

	Total	(%)	34–43 years	44–69 years
HPV positive^**a**^				
Total^a^	3111	100	1507	1604
No cytology or unsatisfactory	229	7.4	120	109
NILM	1278	41.1	545	733
Had repeat HPV test^b^	*626*	49.0^b^	262	*364*
ASC-US	717	23.0	329	388
LSIL	333	10.7	190	143
High-grade^c^	554	17.8	323	231
HPV 16				
Total	650	100	365	285
No cytology or unsatisfactory	49	7.5	25	24
NILM	201	30.9	99	102
Had repeat HPV test^b^	116	57.7^b^	63	53
ASC-US	132	20.3	67	65
LSIL	79	12.2	55	24
High-grade^c^	189	29.1	119	70
HPV 18				
Total	221	100	119	102
No cytology or unsatisfactory	20	9.0	13	7
NILM	74	33.5	33	41
Had repeat HPV test^b^	43	58.1^b^	19	24
ASC-US	53	24.0	29	24
LSIL	22	10.0	11	11
High-grade^c^	52	23.5	33	19
HPV other high risk^d^				
Total	2148	100	975	1173
No cytology or unsatisfactory	150	7.0	77	73
NILM	981	45.7	403	578
Had repeat HPV test^b^	456	46.5^b^	174	282
ASC-US	498	23.2	214	284
LSIL	225	10.5	120	105
High-grade^c^	294	13.7	161	133

*CIN* cervical intraepithelial neoplasia, *NILM* negative for intraepithelial lesion or malignancy, *ASC-US* atypical squamous cells of undetermined significance, *LSIL* low grade squamous intraepithelial lesions.

^a^Total includes 92 (3.0%) women who were not genotyped.

^b^Percentage is calculated as percent of those with NILM test who were positive upon repeat HPV test.

^c^Includes AGC, ASC-H, HSIL, and cancer.

^d^Includes HPV types 31, 33, 35, 39, 45, 51, 52, 56, 58, 59, 66, and 68.

The overall cumulative CIN3+ risk after testing HPV-positive, regardless of cytologic interpretation or HPV genotype, was 23.9% (95%CI = 22.7–24.3%; *n* = 745) and CIN2+ risk was 30.0% (95%CI = 30.1–33.4%; *n* = 964) (data not shown).

### Risks among HPV-positive women referred immediately to colposcopy (ASC-US or worse cytology)

The CIN3+ risk for HPV-positive women with any abnormal cytology, ASC-US or more severe (≥ASC-US) cytology was 38.2% (95%CI = 35.8–40.6%).

CIN3+ and CIN2+ risks for pairwise combinations of HPV and ≥ASC-US categories are shown in Table 2. Women with HPV16-positive high-grade cytology were at the highest risk of CIN3+ (75.7%, 95%CI = 69.4–81.5%) and CIN2+ (81.0%, 95%CI = 75.1–86.2%). By comparison, women with other high-risk HPV-positive ASC-US cytology were at the lowest risk of CIN3+ (18.1%, 95%CI = 15.0–21.7%) and CIN2+ (27.7%, 95%CI = 24.0–31.9%).

**Table 2:** Risk of CIN3+ and CIN2+ among HPV-positive women by ≥ASC-US cytology and HPV genotype.

		CIN3+				CIN 2+			
	Total	*N* (cases)	Risk	95%CI		*N* (cases)	Risk	95%CI	
All HPV positive^a^									
ASC-US	717	154	21.5	18.7	24.7	223	31.1	27.9	34.6
LSIL	333	99	29.7	25.2	35.0	146	43.8	38.7	49.4
High-grade^b^	554	359	64.8	60.9	68.8	397	71.7	67.9	75.4
≥ASC-US	1604	612	38.2	35.8	40.6	766	47.8	45.3	50.2
HPV16									
ASC-US	132	51	38.6	30.9	47.5	63	47.7	39.6	56.6
LSIL	79	37	46.8	36.6	58.4	49	62.0	51.5	72.6
High-grade	189	143	75.7	69.4	81.5	153	81.0	75.1	86.2
≥ASC-US	400	231	57.8	53.0	62.6	265	66.3	61.6	70.9
HPV18									
ASC-US	53	11	20.8	12.1	34.3	18	34.0	22.9	48.4
LSIL	22	6	27.3	13.3	50.9	11	50.0	31.6	71.8
High-grade	52	34	65.4	52.6	77.9	38	73.1	60.7	84.2
≥ASC-US	127	51	40.2	32.3	49.2	67	52.8	44.4	61.6
Other high-risk HPV^**c**^									
ASC-US	498	90	18.1	15.0	21.7	138	27.7	24.0	31.9
LSIL	225	55	24.4	19.4	30.6	84	37.3	31.4	44.0
High-grade	294	174	59.2	53.6	64.8	198	67.4	62.0	72.6
≥ASC-US	1017	319	31.4	28.7	34.3	420	41.3	38.4	44.4

*CIN* cervical intraepithelial neoplasia, *CI* confidence interval, *ASC-US* atypical squamous cells of undetermined significance, *LSIL* low grade squamous intraepithelial lesions.

^a^Includes all HPV-positive persons with ≥ ASC-US cytology, including 60 not HPV genotyped.

^b^High grade includes AGC, ASC-H, HSIL, and cancer.

^c^Includes HPV types 31, 33, 35, 39, 45, 51, 52, 56, 58, 59, 66, and 68.

**Fig. 2:**
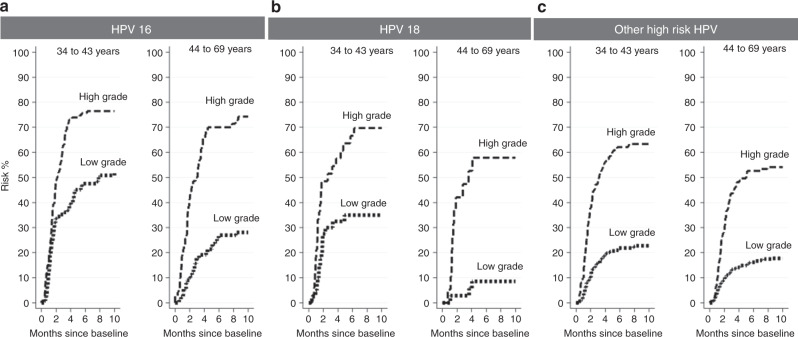
Risk of CIN3+ by low-grade and high-grade cytology for 34–43 and 44–69 years old women by HPV genotype. (**a**) HPV16 genotype, (**b**) HPV18 genotype, (**c)** other high-risk HPV genotype. High grade: AGC, ASC-H, HSIL, ACIS, and SCC; Low grade: ASC-US and LSIL; Other high-risk HPV genotype includes 31, 33, 35, 39, 45, 51, 52, 56, 58, 59, 66, 68. Abbreviations: ASC-US, atypical squamous cells of undetermined significance; LSIL, low-grade squamous intraepithelial lesions, AGC, atypical glandular cells; ASC-H, atypical squamous cells, cannot rule out high-grade squamous intraepithelial lesion; HSIL, high-grade squamous intraepithelial lesions; ACIS; atypical glandular cells Cervical adenocarcinoma in situ; SCC, squamous cell carcinoma.

Stratified on the median screening age of 44 years (IQR: 38–52 years), overall CIN3+ risk was higher for younger women (34–43 years) (30.6%; 95%CI = 28.3–33.2%; *n* = 457) compared to older women (44–69 years) (18.1%; 95%CI = 16.3–20.9%; *n* = 288) (*P* < 0.001). Figure 2 show risks of CIN3+ for HPV genotype and high-grade versus low-grade cytology. Within each age group, higher risk HPV and more severe cytologic categories independently increased the CIN3+ risk. Younger women were at higher CIN3+ risk than older women but the differences were only significant for all HPV-positive ASC-US (27.7%, vs. 16.2%, *P* < 0.001) and HPV16-positive ASC-US (47.8%, vs 29.2%, *P* = 0.02) and LSIL (35.2%, vs. 26.1%, *P* = 0.01) (data not shown).

### Risks among HPV-positive women with NILM cytology

Women positive for HPV16/18 with NILM cytology had higher CIN3+ risk compared to women positive for other high-risk HPV with NILM cytology. CIN3+ risk by HPV genotype for NILM were 19.9% (95%CI = 15.0–26.1%) for HPV16, 10.8% (95%CI = 5.6–20.5%) for HPV18 and 5.5% (95%CI =  4.2–7.1%) for other high-risk HPV (Table 3).

**Table 3:** Risk of CIN3+ and CIN2+ among HPV-positive women with NILM cytology by HPV genotype at baseline screening until regular follow-up of 21 months.

		CIN3+				CIN2+			
	Total	*N* (cases)	Risk	95%CI		*N* (cases)	Risk	95%CI	
Baseline HPV Result									
HPV-positive^a^	1278	102	8.0	6.6	9.6	154	12.1	10.4	14.0
HPV16	201	40	19.9	15.0	26.1	49	24.4	19.0	30.9
HPV18	74	8	10.8	5.6	20.5	12	16.2	9.6	26.8
Other high-risk HPV^b^	981	54	5.5	4.2	7.1	92	9.4	7.7	11.4

*CIN* cervical intraepithelial neoplasia, *CI* confidence interval, *NILM* negative for intraepithelial lesion or malignancy.

^a^Includes all HPV-positive persons with NILM cytology, including 22 not HPV genotyped.

^b^Includes HPV types 31, 33, 35, 39, 45, 51, 52, 56, 58, 59, 66, and 68.

Among those women with HPV-positive and NILM cytology results, 626 (49.0%) re-tested HPV positive in a year. Among those who repeatedly tested HPV-positive, most (539 of 564, 95.6%) were positive for the same HPV genotype. Twelve-month CIN3+ risks were 29.5% (95%CI = 21.8–39.3%) among women who twice tested HPV16 positive, 15.6% (95%CI = 6.8–33.5%) among women who twice tested HPV18 positive, and 10.2% (95%CI = 7.7–13.6%) among women who twice tested positive for other high-risk HPV (Table 4). By comparison, the CIN3+ risk for twice testing high-risk HPV positive, regardless of specific HPV results, was 14.0% (95%CI = 11.4–17.2%).

**Table 4:** Risk of CIN3 by second HPV genotype 12 months after a baseline screening result for HPV-positive and NILM cytology. Concordant results by HPV genotype are in italic.

	12-month-year follow-up HPV test results															
	HPV16				HPV18				Other high-risk HPV				Any high-risk HPV (HPV16, 18, and other high risk)			
Baseline HPV test results	*N* (cases)	*N*	Risk	95%CI	*N* (cases)	*N*	Risk	95%CI	*N* (cases)	*N*	Risk	95%CI	*N* (cases)	*N*	Risk	95%CI
HPV16	*31*	*105*	*29.5*	*21.8–39.3*	0	0	–^c^	– ^c^	0	4	–^c^	–^c^	31	109	28.4	21.9–37.9
HPV18	0	0	–^c^	– ^c^	*5*	*32*	*15.6*	*6.8–33.5*	1	4	25.0	4.0–87.2	6	36	16.7	7.9–33.4
Other high-risk HPV	0	12	– ^c^	– ^c^	1	5	20.0	3.1–79.6	*41*	*402*	*10.2*	*7.6–13.6*	42	419	10.0	7.5–13.3
Any high-risk HPV	31	117	26.5	19.4–35.5	6	37	16.2	7.6–32.6	42	410	10.2	7.7–13.6	*79*^a^	*564*^b^	*14.0*	*11.4–17.2*

*CIN* cervical intraepithelial neoplasia, *CI* confidence interval, *NILM* negative for intraepithelial lesion or malignancy.

^a^Excludes 62 individuals with missing genotype data out of 626 (9.9%) individuals who were HPV positive a second time after baseline HPV-positive and NILM results.

^b^Excludes 9 individuals with missing genotype data out of 88 (0.1%) individuals who were HPV positive a second time after baseline HPV-positive and NILM results.

^c^Sample size insufficient to yield meaningful results.

**Fig. 3:**
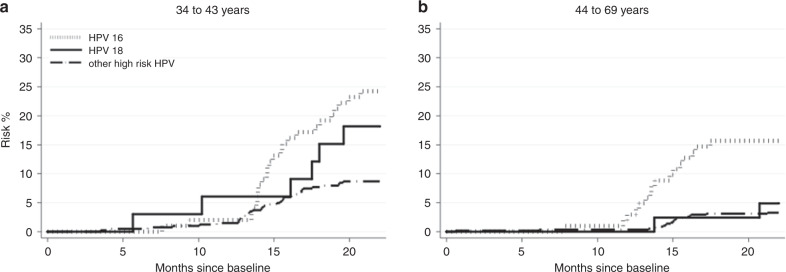
Risk of CIN3+ by NILM cytology stratified by genotype. (**a**) 34–43-year-old women (**b**) 44–69-year-old women. Abbreviations: NILM, negative for intraepithelial lesion or malignancy. Other high-risk HPV includes 31, 33, 35, 39, 45, 51, 52, 56, 58, 59, 66, 68.

Among women with an HPV-positive/NILM result at baseline, those aged 34–43 years had a higher CIN3+ risk overall (11.9%; 95%CI = 9.1–14.0%) than women aged 44–69 years (4.8%; 95%CI = 3.4–6.7%) (*P* < 0.001) (Figure 3 and Supplementary Table 1). By HPV genotype, the risk between the two age groups were different for other high-risk HPV only (*P* < 0.001). When stratified for age, the results among HPV genotypes upon second HPV testing also were only significantly different among women who were overall HPV positive and women (*P* < 0.001) who were other high-risk HPV positive (*P* < 0.001). All other persistent HPV genotypes had *P*-values greater than 0.05 (data not shown).

## Discussion

For combinations of HPV-genotype and cytology results using real-world screening data in Norway, women who tested positive for HPV16 and/or HPV18 genotype had a higher risk of CIN3+ and CIN2+ compared with women who tested positive for other high-risk. This was found across every cytologic result. Women, who tested positive for HPV16 and/or HPV18 and ≥ASC-US had a higher risk for CIN3+ and CIN2+, and should be referred for immediate colposcopy. The benefits and harms of managing women based on HPV positivity and cytology results can be better balanced by managing women with other high-risk HPV (not HPV16 or HPV18) and low-grade cytology (ASC-US or LSIL) with surveillance.

In Norway, cervical cancer management has been based on results from cervical cytology primary screening.^28^ The switch to the more sensitive HPV test as the primary screening test can lead to over-referral to colposcopy, again leading to increased likelihood of morbidity.^43–45^ Although an HPV positive result alone does confer a high risk of cervical precancer and cancer compared to a negative result, not all HPV genotypes are equally likely to persist^46^ or develop into precancer or cancer.^10^

To increase the accuracy of the HPV screening, a cytological evaluation of HPV positives with divergent clinical management at threshold ASC-US+ has been recommended internationally. Although the Bethesda classification provide criteria for cytological definitions universally, systematic differences between pathology reviewers and countries has been reported in replicating cytological results.^47^ These differences might explain variation in reported proportion of those with ASC-US+ cytology (range 27–46%).^48–50^ Inter-laboratory differences for cytology results, are commonly observed in Norway, suggesting further that locally agreed diagnostic criteria and routines are applied.^51^ HPV testing in screening, however, demonstrated an excellent agreement between labs,^24^ suggesting wider application of molecular testing in the follow-up. Our study demonstrate that HPV genotyping provides further risk stratification and reduces an over-referral of all women who have an HPV positive and low-grade or NILM cytology result to colposcopy and biopsy.

To avoid an over-referral of all women who have an HPV positive and low-grade or NILM cytology result to colposcopy and biopsy, HPV genotyping provides further risk stratification.

The present analysis of HPV genotypes show consistent results with the POBASCAM and ATHENA trials, which showed higher precancer and cancer risk among HPV16/18 positive women (without cytology testing).^34,52,53^ With cytology testing included, the Portland-Kaiser study, a large cohort of ~20,000 women, found that HPV16 had a 2.7 times higher risk of precancer and cancer compared to other HPV genotypes across all cytology results.^54^

Among women with baseline cytology of NILM and an HPV genotype of HPV16, 18, or other high-risk HPV type, women who continue to test positive for the same HPV genotype upon two screening rounds had an elevated risk compared to those with whole sub-cohort of women with HPV-positive and NILM cytology result at baseline. CIN3+ risk was highest for women with persistent HPV16 positive results (30%) and lowest for persistent other high-risk HPV-positive results (10%), indicating that the most of the 14% CIN3+ risk for women with NILM cytology and repeat HPV-positive test is due to persistent HPV16 infection. Similarly, an English pilot study found that HPV 16/18 triage of persistently high-risk HPV-positive and cytological negative women 12 months after primary screening added very little in terms of a clinical benefit such as additional detection of CIN2+ after baseline HPV genotyping.^55^ It must be noted that, in this analysis, sample sizes among women testing for two different HPV genotypes upon repeat testing were small [i.e. HPV18 followed up by other high-risk HPV positivity (*n* = 4)]. Thus, these findings require replication in larger populations.

The conclusions drawn from the genotype results for the Norwegian HPV screening implementation provide the basis for “equal management for equal benchmark risk” approach as performed in the US and certain European countries. This risk-based approach^12,56^ uses a screening (HPV genotype and cytology) result as a benchmark for clinical management decisions for HPV-positive women. Based on real-world screening results from the NCCSP, the risk threshold for a colposcopy referral among HPV-positive women has been equivalent to the risk for other high-risk HPV and LSIL result or ~37.3% for CIN2+ and 24.4% for CIN3+. Women above this threshold, such as those with high-grade cytologic abnormalities or low-grade cytologic abnormalities and test HPV16 or HPV18 positive, should immediately be referred to colposcopy and biopsy. Women with one of these risk markers, other high-risk HPV positive with low-grade cytologic abnormalities or test HPV16 or HPV18 positive with NILM have a risk for CIN3+ below 24.4% and can be re-tested in 12 months. Women with neither marker, i.e., NILM cytology and positive for other high-risk HPV, can be retested in 24 months as they have less than half the precancer and cancer risk of women with one of these risk markers.

This analysis focuses on immediate risks of CIN3+ by HPV genotype, including results for women with HPV+ and NILM cytology on their baseline test that are retested after one year. The negative predictive value of this follow-up regimen can be confirmed in long-term follow-up only. The relative rank order between the risk groups observed in this study is consistent with those HPV genotype/cytology screening results reported elsewhere.^12,56^ It must also be noted that the threshold at which the risk of cancer in an individual patient outweighs any disadvantage of management and treatment varies according to the patient’s characteristics and local service considerations. In a country like Norway, with an established screening program and with high follow-up of screen positives, the threshold for colposcopy referral and treatment may be set higher safely, due to the relatively low risk among women with negative test results. Taking these factors under consideration, these results should be interpreted as a basis for the incorporation of HPV genotyping into primary screening as well as a rationale for risk reductions following over-referral of women to colposcopy and biopsy after primary HPV screening using real-world screening data.

There were several limitations. First, there was only a short follow-up time for this cohort. Second, estimates of cervical disease (CIN2+ and CIN3+) were calculated based on women who underwent concurrent colposcopy and biopsy. Women with an NILM cytology and HPV-negative result are not typically referred to colposcopy and biopsy and therefore, their cervical histology status is not known. Women who do undergo immediate colposcopy and biopsy with an NILM cytology result, which is against NCCSP and most other cervical cancer screening guidelines, likely represent a biased sample.^57^ It has also been shown that precancer and cancer risk for women with an NILM cytology and HPV-positive result is 5.9%, making the likelihood of undetected precancer and cancer in a subset of these women quite low.^49^

Despite these limitations, this study has notable strengths. This is the first study to investigate the role of HPV partial genotyping and cytology in an HPV-based screening programme in Norway. Norway has a population-based registration system of cytology results and biopsies and all results are included even if women chose to attend private healthcare. This system reduces loss to follow-up risk and selection biases and has shown to be highly reproducible.^58^ Further, data were collected uniformly, and laboratory tests were performed within an organised cervical cancer screening program with relatively high-population coverage. Results of this analysis can, therefore, be generalisable to a larger population-based cervical screening program, which is a prerequisite for advocating population-wide cancer screening management by HPV genotype. While not generalisable to certain other populations with different patterns of care, compliance, testing, rescreening intervals, or risk factors,^12^ the results of this study have altered the management protocol for HPV-positive women living in Norway,^59^ providing an example of using population-based screening data as a basis for which to implement screening (Supplementary Fig. 2). The results obtained are thus valuable for both Norwegian and non-Norwegian policymakers, as well as for public health organisations that aim to reduce cervical cancer burden.

In conclusion, we found that HPV16 and HPV18 have a higher risk for CIN3+ and CIN2+ and partial HPV genotyping, in combination with cytology results, can better differentiate HPV-positive women at higher and lower risk for cervical precancer or cancer than cytology alone. This is crucial for women who are at too high risk for surveillance alone, such as those with HPV16/18 and low-grade cytology, while limiting unnecessary colposcopy and biopsy for women with lower precancer or cancer risk, such as those positive for other high-risk HPV genotypes and low-grade cytology. These findings provided the evidence for and adoption of the use of partial HPV genotyping for the management of HPV-positive women aged 34–69 years in Norway.^59^

## Supplementary information

Supplementary material

## Data Availability

All data not included in this published article are available upon reasonable request from the Cancer Registry of Norway.
