# Supplementary material for: Real-world data on cervical cancer risk stratification by cytology and HPV genotype to inform the management of HPV-positive women in routine cervical screening
Source: Br J Cancer. 2020 Apr 3;122(11):1715–23. doi: 10.1038/s41416-020-0790-1 (PMC7250848; doi:10.1038/s41416-020-0790-1)
Supplement: Supplementary file 1 — Supplementary material [file 41416_2020_790_MOESM1_ESM.docx]

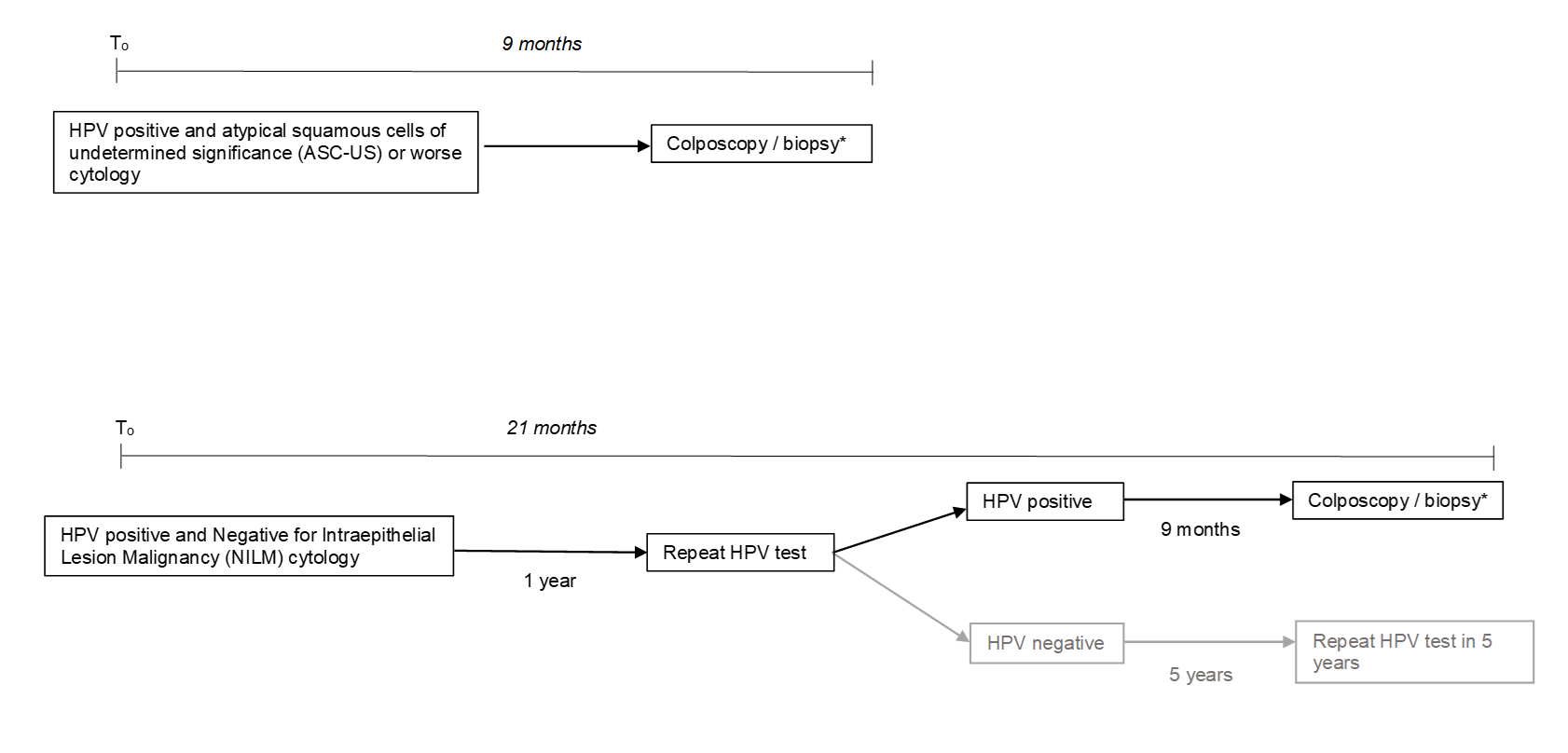


Supplemental Figure 1. Flow diagram describing clinical guideline recommendations for HPV-positive participants at T0 in the Norwegian Cervical Cancer

Screening Program, 2015 to 2018. *All biopsies or treatments taken within 9 months after baseline (T0) were defined as immediate colposcopy/biopsy.

| Supplementary Table 1. Risk of CIN3+ among HPV-positive women with NILM cytology by HPV genotype at baseline screening until regular follow-up by age group | | | | | | | | | | | | |
| --- | --- | --- | --- | --- | --- | --- | --- | --- | --- | --- | --- | --- |
|  |  |  |  |  |  |  |  |  |  |  |  |  |
|  | 34 to 43 years old | | | | |  | 44 to 69 years old | | |  |  |  |
| HPV genotype | N(cases) | N | Risk | 95%CI | |  | N(cases) | N | Risk | 95%CI | | P value |
|  |  |  |  |  |  |  |  |  |  |  |  |  |
| All HPV positives^a^ | 65 | 545 | 11,9 | 9,1 | 14,0 |  | 35 | 733 | 4,8 | 3,4 | 6,7 | <0.001 |
| HPV16 | 24 | 99 | 24,2 | 15,2 | 30,8 |  | 16 | 102 | 15,7 | 11,2 | 27,1 | 0.60 |
| HPV18 | 6 | 33 | 18,2 | 7,4 | 31,8 |  | 2 | 41 | 4,9 | 1,4 | 20,4 | 0.14 |
| Other high risk HPV^b^ | 35 | 403 | 8,7 | 6,5 | 11,7 |  | 21 | 578 | 3,6 | 1,5 | 4,3 | <0.001 |

^a^Includes all HPV-positive persons with NILM cytology, including 22 not HPV genotyped.

^b^Includes HPV types 31, 33, 35, 39, 45, 51, 52, 56, 58, 59, 66, and 68.

CIN, cervical intraepithelial neoplasia; CI, confidence interval; NILM, negative for intraepithelial lesion or malignancy.


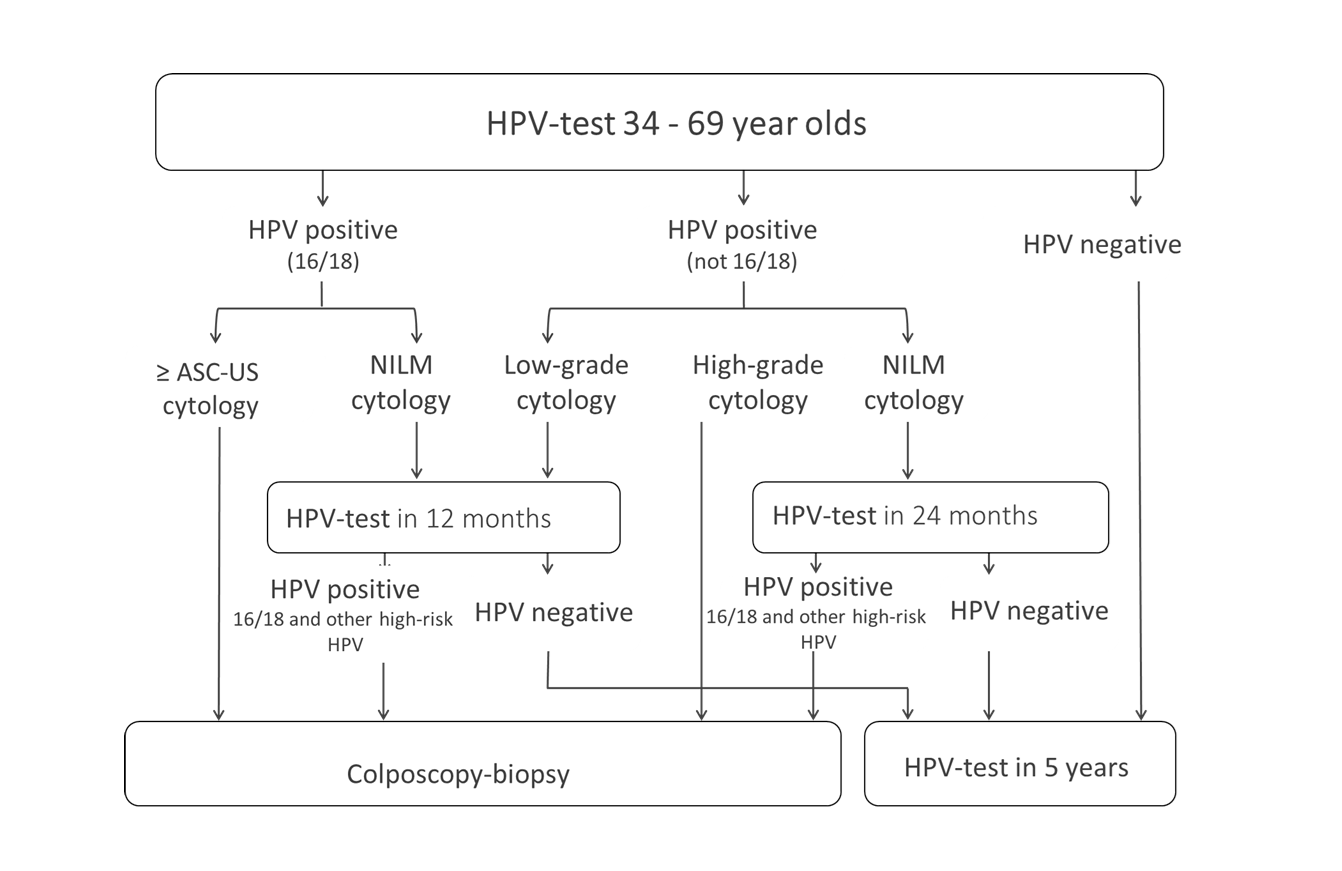


Supplemental Figure 2. New clinical guideline recommendations for HPV-positive women aged 34 to 69 years in the Norwegian Cervical Cancer Screening

Program, July 1st 2018.

Abbreviations: NILM, negative for intraepithelial lesion or malignancy; ASC-US, atypical squamous cells of undetermined significance; LSIL, low-grade

squamous intraepithelial lesions, AGC, atypical glandular cells; ASC-H, Atypical squamous cells, cannot rule out high-grade squamous intra-epithelial lesion;

HSIL, high-grade squamous intraepithelial lesions; ACIS; Atypical glandular cells cervical adenocarcinoma in situ. High-grade refers to AGH, ASC-H, HSIL, ACIS,

and cancer. Low-grade refers to ASC-US and LSIL.
